# Ability of ^18^F-FDG Positron Emission Tomography Radiomics and Machine Learning in Predicting KRAS Mutation Status in Therapy-Naive Lung Adenocarcinoma

**DOI:** 10.3390/cancers15143684

**Published:** 2023-07-19

**Authors:** Ruiyun Zhang, Kuangyu Shi, Wolfgang Hohenforst-Schmidt, Claus Steppert, Zsolt Sziklavari, Christian Schmidkonz, Armin Atzinger, Arndt Hartmann, Michael Vieth, Stefan Förster

**Affiliations:** 1 Institute of Pathology, Medizincampus Oberfranken, Klinikum Bayreuth, Friedrich-Alexander-Universität Erlangen-Nürnberg, 95445 Bayreuth, Germany; 2 Institute of Pathology, Universitätsklinikum Erlangen, Friedrich-Alexander-Universität Erlangen-Nürnberg, 91054 Erlangen, Germany; 3 Department of Nuclear Medicine, Inselspital Bern, 3010 Bern, Switzerland; 4 Department of Pneumology, Sana Klinikum Hof, 95032 Hof, Germany; 5 Department of Pneumology, REGIOMED Klinikum Coburg, 96450 Coburg, Germany; 6 Department of Thoracic Surgery, Klinikum Coburg, 96450 Coburg, Germany; 7 Department of Nuclear Medicine, Universitätsklinikum Erlangen, Friedrich-Alexander-Universität Erlangen-Nürnberg, 91054 Erlangen, Germany; 8 Department of Nuclear Medicine, Klinikum Bayreuth, 95445 Bayreuth, Germany; 9 Medizincampus Oberfranken, Universitätsklinikum Erlangen, 95445 Bayreuth, Germany; 10 Department of Nuclear Medicine, Klinikum rechts der Isar der Technischen Universitaet Muenchen, 81675 München, Germany

**Keywords:** PET, KRAS, machine learning, lung adenocarcinoma, radiomic features

## Abstract

**Simple Summary:**

Approximately 26.1% of patients diagnosed with lung adenocarcinoma harbour a KRAS mutation, which is associated with a poorer prognosis. Recent advances in targeted therapy, specifically with sotorasib and MRTX849, have shown promise in targeting KRAS mutations. This retrospective study aimed to develop a clinical prediction model that combines clinical–pathological variables and radiomics derived from PET scans to assess the KRAS mutation status in patients with lung adenocarcinoma. This study utilised two different databases and randomly divided into a training, a validation, and a testing dataset to build and evaluate the predictive performance of our model. Our retrospectively developed model demonstrates good predictive accuracy for determining the KRAS mutation status in lung adenocarcinoma patients.

**Abstract:**

Objective: Considering the essential role of KRAS mutation in NSCLC and the limited experience of PET radiomic features in KRAS mutation, a prediction model was built in our current analysis. Our model aims to evaluate the status of KRAS mutants in lung adenocarcinoma by combining PET radiomics and machine learning. Method: Patients were retrospectively selected from our database and screened from the NSCLC radiogenomic dataset from TCIA. The dataset was randomly divided into three subgroups. Two open-source software programs, 3D Slicer and Python, were used to segment lung tumours and extract radiomic features from ^18^F-FDG-PET images. Feature selection was performed by the Mann–Whitney U test, Spearman’s rank correlation coefficient, and RFE. Logistic regression was used to build the prediction models. AUCs from ROCs were used to compare the predictive abilities of the models. Calibration plots were obtained to examine the agreements of observed and predictive values in the validation and testing groups. DCA curves were performed to check the clinical impact of the best model. Finally, a nomogram was obtained to present the selected model. Results: One hundred and nineteen patients with lung adenocarcinoma were included in our study. The whole group was divided into three datasets: a training set (*n* = 96), a validation set (*n* = 11), and a testing set (*n* = 12). In total, 1781 radiomic features were extracted from PET images. One hundred sixty-three predictive models were established according to each original feature group and their combinations. After model comparison and selection, one model, including wHLH_fo_IR, wHLH_glrlm_SRHGLE, wHLH_glszm_SAHGLE, and smoking habits, was validated with the highest predictive value. The model obtained AUCs of 0.731 (95% CI: 0.619~0.843), 0.750 (95% CI: 0.248~1.000), and 0.750 (95% CI: 0.448~1.000) in the training set, the validation set and the testing set, respectively. Results from calibration plots in validation and testing groups indicated that there was no departure between observed and predictive values in the two datasets (*p* = 0.377 and 0.861, respectively). Conclusions: Our model combining ^18^F-FDG-PET radiomics and machine learning indicated a good predictive ability of KRAS status in lung adenocarcinoma. It may be a helpful non-invasive method to screen the KRAS mutation status of heterogenous lung adenocarcinoma before selected biopsy sampling.

## 1. Introduction

In the 2020 cancer statistics, lung cancer had the highest morbidity and mortality of cancer-causing death in men and was the third highest for incidence and second highest for mortality in women [1]. There are two main entities in lung cancer: non-small cell lung cancer (NSCLC), accounting for about 85%, and small cell lung cancer (SCLC), accounting for about 15%; adenocarcinoma is the most frequent NSCLC.

Recently, the treatment paradigm has been changed since tyrosine kinase inhibitors (TKI) targeting epidermal growth factor receptors (EGFRs) have been used in NSCLC [2]. Better outcomes were seen in patients with NSCLC treated with TKI targeting the anaplastic lymphoma kinase gene (ALK) [3], EGFR [4], and ROS1 [5]. Kirsten rat sarcoma viral oncogene homolog (KRAS) was also tested in NSCLC and occurs in about 26.1% of lung adenocarcinoma in Western countries [6]. But KRAS was considered undruggable for quite a long period, and tumours with KRAS mutation tended to have a worse outcome [7,8]. Recently, sotorasib and MRTX849 showed prolonged survival time and fewer adverse effects in patients with KRAS-driven NSCLC [9,10], indicating that TKI therapy targeting KRAS may become more critical, and, therefore, testing for KRAS mutant status before selecting a specific treatment may also become more critical.

In previous studies for NSCLC, smoking history and male gender were reported to be highly correlated with KRAS mutation status [11,12,13,14,15,16,17], but the results are still controversial. Several studies analysed the relationship between PET/CT conventional parameters and the mutational status of KRAS. Their results indicate that higher SUVmax is correlated with KRAS-positive status in colorectal cancer and pancreatic ductal adenocarcinoma [18,19,20,21,22], but in similar studies for NSCLC, this trend was not found in most of the previous studies [23,24,25], except in two [7,8].

Radiomics were proposed by Lambin in 2012 [26], aiming to extract a huge number of advanced features from medical images. Combined with machine learning, radiomics showed better ability in distinguishing benign and malignant tumours and predicting gene mutation status and treatment results than clinicopathological variables and conventional PET/CT parameters. Based on these experiences, radiomics from CT or PET/CT were examined to determine whether they are able to predict KRAS mutation status [25,27,28,29]. These results demonstrated a promising trend in evaluating KRAS mutation. Unfortunately, the number of studies is insufficient to obtain a robust conclusion, and the experience from PET radiomics is still limited.

Our present study combined FDG-PET radiomics with machine learning to establish and validate a clinical prediction model evaluating KRAS mutation status in lung adenocarcinoma.

## 2. Method and Materials

### 2.1. Patients Selection

The patients’ datasets were selected from two different resources: the Klinikum Bayreuth database and The Cancer Imaging Archive (TCIA) [30]. For the samples from Klinikum Bayreuth, patient informed consent was obtained before the ^18^F-FDG PET/CT scan. Retrospective data analysis was approved by the Ethics Committee of the University of Erlangen-Nuremberg (Application No.: 430_19 Bc. ClinicalTrials.gov Identifier: NCT04276025). Related to the datasets from TCIA, ethical approval and informed consent were not required.

Inclusion criteria: (1) lung adenocarcinoma diagnosed histologically; (2) no previous malignant tumour history; (3) no antitumor treatments before PET scan; (4) blood glucose level (BGL) below 150 mg/dl before ^18^F-FDG injection; and (5) available result of KRAS mutation analysis. Exclusion criteria: the quality of the PET image was not good enough to extract radiomic features. Based on these criteria, eligible patients were selected from 1 June 2016 to 31 December 2020 from the Klinikum Bayreuth database and 211 samples from the Lung Radiogenomic database from TCIA.

The patients included in our study were randomly divided into three groups: one training dataset, one validation dataset, and one testing dataset. First, 10% of the samples were randomly chosen from the whole dataset as a testing group; second, another 10% of the samples were selected from the leftover dataset as the validation group; finally, the rest of the leftover samples were assigned to the training dataset.

### 2.2. Detection of KRAS Mutation

*In our dataset.* The KRAS mutation status was recorded directly from the electronic medical system. 

*In the dataset from TCIA.* Single nucleotide mutation detection was performed using SNaPshot technology based on dideoxy single-base extension of oligonucleotide primers after multiplex PCR.

### 2.3. Image Acquisition

*In our dataset.* The PET/CT scanning of all patients was performed in Klinikum Bayreuth. Siemens Biograph mCT (Erlangen, Germany) was used. All patients fasted for 6–8 h before PET/CT scan. About 1 h after injection, the images were acquired. At first, low-dose CT was performed at tube current of 30 mAs and tube voltage of 120 kV. The PET images were corrected using low-dose CT and reconstructed iteratively (2 iterations, 12 subsets) + TrueX + TOF.

*For The dataset from TCIA.* GE Discovery D690 PET/CT and GE Discovery STE PET/CT scanners were used. The mean uptake time before scanning was 66.58 min (range 23.08~128.90 min). Low-dose CT was performed, tube current 36~400 mAs, tube voltage 120~140 kV. The PET images were corrected using CT images based on iterative Ordered Subset Expectation Maximization (OSEM) reconstruction.

### 2.4. Tumour Segmentation and Radiomic Features Extraction

*Tumour segmentation.* An open-source software 3D Slicer (Version 4.11) was used to segment the tumour. The “PET-IndiC” extension was installed, and the “PETTumorSegmentationEffect” tool was used to define the region of interest (ROI) automatically. After an ROI was determined, the conventional parameters, including SUVmax and TLG, were calculated automatically. The original images and labels were exported in “.nrrd” format.

*Image pre-processing.* Textural feature extraction was performed in Python (Version 3.7.4). The related Python libraries were as follows: Pyradiomics (Version 3.0.1), Numpy (Version 1.20.3), and PyWavelet (Version 1.1.1). Before feature extraction, all images were interpolated with the sitkBSpline algorithm. The PET images were resampled as 3*3*3 mm^3^, and “binWidth” was set as 64 [25].

*Feature extraction.* In total, 1781 radiomic features were obtained. These features were from (1) original radiomic features (107 features): shape-based (14 features), first-order (18 features), GLCM (24 features), GLDM (14 features), GLRLM (16 features), GLZLM (16 features), and NGTDM features (5 features); (2) Wavelet-transformed features (744 features): transformation for first-order and textural features in eight directions (LLH, LHL, LHH, HLL, HLH, HHL, HHH, and LLL); and (3) Laplacian of Gaussian (LOG) features (930 features), also applied on first-order and textural features according to ten different sigma values (from 0.5 to 5, with step 0.5).

### 2.5. Feature Selection

Several methods and steps were used for the selection: (1) observation: some features were normalised during feature extraction; we removed such kinds of features first before taking further steps; (2) Mann–Whitney U test: only features with *p* < 0.05 were kept; (3) Spearman’s rank correlation coefficient: when ρ > 0.8 and *p* < 0.05 were found between two features, the one with higher *p* value in U test was deleted; and (4) RFE ensembled with RF: before RFE, the kept features from (3) were normalised using Z-score normalisation.

Considering the differences between the subgroups of Wavelet and LOG features, we defined our features into 23 groups, namely clinicopathological variables, metabolic PET parameters, original radiomic features, eleven feature groups from LOG (log, log05, log1, log15, log2, log25, log3, log35, log4, log45, and log5), and nine groups from Wavelet (Wavelet, wLLH, wLHH, wLHL, wHLH, wHHL, wHLL, wHHH, and wLLL). These feature groups were combined randomly except for the subgroups from LOG and Wavelet. One hundred sixty-three different model groups were analysed in the end. 

In the model based on clinicopathological variables, metabolic PET parameters, and their combination, only RFE was performed. For the remaining 160 models, steps 2 to 4 were taken.

### 2.6. Predictive Model Building and Evaluation

*Model building.* Logistic regression was used to establish prediction models using the features after feature selection.

*Model evaluation.* Limited by the small sample size, five-fold cross-validation 5-fold CV was performed first on the training dataset. Models without overfitting in 5-fold CV were tested by the validation set. They were applied to the testing set if a model did not show any overfitting or underfitting in the validation group. AUCs from ROCs were used to compare the models that showed predictive abilities in 5-fold CV, validation, and testing sets. Finally, the best model was chosen. Calibration plots in validation and testing, DCA curves in three datasets, and nomogram in the train set were plotted based on the best model.

### 2.7. Statistical Analysis

The analysis was performed in two open-source software: Python (Version 3.7.4) and R language (Version 3.6.3).

*In Python,* dataset split and data standard normalisation were performed using scikit-learn (sklearn, Version 0.24.2); the Mann–Whitney U test and Spearman’s rank correlation coefficient were performed by scipy (Version 1.6.3).

*In R,* package “caret” was used to perform RFE, repeated cross-validation, and establish logistic regression; “reportROC” was used to obtain ROCs; calibration curves and nomogram were plotted by package “rms”; and package “rmda” was used to obtain decision curve analysis (DCA).

## 3. Results

### 3.1. Clinicopathological Variables in Different Groups

According to the inclusion and exclusion criteria, 10 patients from Klinikum Bayreuth and 109 patients from TCIA were eligible for our study. 

*Klinikum Bayreuth dataset.* The results of KRAS mutation in these ten patients were all positive. In this group, there were three males and seven females, the median age was 68.5 years (range 58 to 85 years), and all of them had a history of smoking.

*TCIA dataset.* There were 27 cases with KRAS mutant status and 82 cases with wild-type KRAS status. The dataset included 65 males and 44 females; the median age was 68 years (range 24 to 87 years). Among these, 30 cases were non-smokers, and 79 cases had a history of smoking.

*Dataset description.* After a random split, we obtained three datasets: a training set (*n* = 96, with 30 KRAS mutant and 66 KRAS wild type); a validation set (*n* = 11, with three KRAS mutant and eight KRAS wild type); and a testing set (*n* = 12, with four KRAS mutant and eight KRAS wild type). The detailed information and the univariate analysis results are presented in Table 1.

### 3.2. Feature Selection and Model Establishment

There were four steps in feature selection. In the whole dataset, we found 620 radiomic features, which were normalised after feature extraction and were removed before further steps. Finally, five clinicopathological variables, two conventional PET parameters, and 1161 radiomic features were included. In the 163 models, after Spearman’s rank correlation coefficient, we found 62 models were duplicated with others; they were removed before RFE.

Finally, 101 predictive models were built based on logistic regression after feature selection.

### 3.3. Model Comparison and Selection

All models showed predictive ability in the training set (AUC range: 0.67 to 0.95). In the five-fold CV, 75 models showed overfitting. Twelve models showed underfitting, and four models showed overfitting in validation groups. Finally, ten models were tested in the testing dataset. Among those, seven models detected overfitting.

The features of the remaining three models were from five different groups: clinical variable, PET metabolic parameters, original radiomic features, wHLH and wLHL. Next, we analysed all possible combinations of these five groups. In the end, four new models were obtained. In the training set, they all had good predictive abilities (AUC range: 0.76 to 0.80) but showed overfitting in 5-fold CV (AUC range: 0.54 to 0.66).

Three models were selected for the final comparison. Model 1: clinical model, including diameter and smoking history; model 2: clinical variables + metabolic parameters + wHLH model, including smoking history, wHLH_fo_IR, wHLH_glrlm_SRHGLE, and wHLH_glszm_SAHGLE; and model 3: clinical variables + original radiomics + wLHL model, including gender, wLHL_fo_Variance, and original_glrlm_SRE. The comparison results are presented in Table 2.

**Figure 1:**
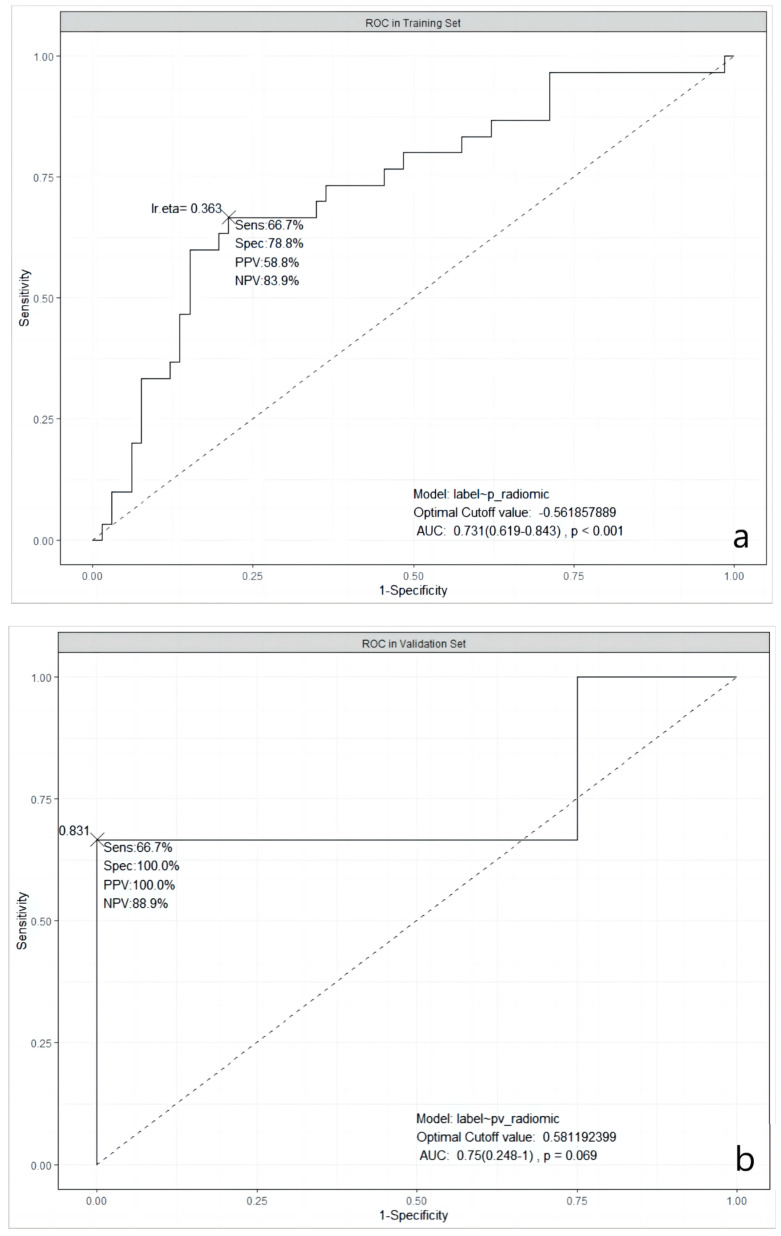

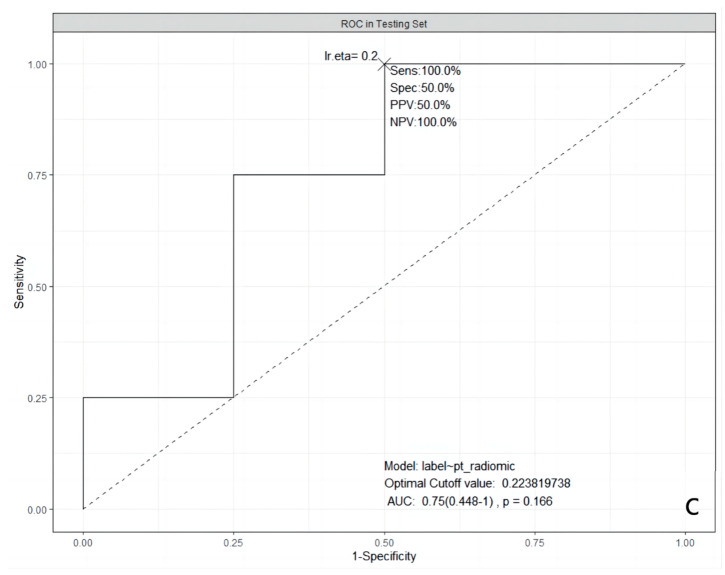
ROC curves of the selected model in three datasets. (**a**): in the training dataset; (**b**): in the validation dataset; and (**c**): in the testing dataset. ROC: Receiver Operating Characteristic; AUC: Area Under the ROC Curve; Sens: Sensitivity; Spec: Specificity; PPV: Positive Predictive Value; NPV: Negative Predictive Value; lr.eta: Learning Rate eta.

It was obvious that the predictive abilities of model 2 were higher than others, no matter in the training set, 5-fold CV, validation, or testing set. In the end, model 2 was chosen as the best model in our study. The ROC curves of model 2 in the training, the validation, and the testing sets are shown in Figure 1.

### 3.4. Calibration Plots in Validation and Testing Groups

**Figure 2:**
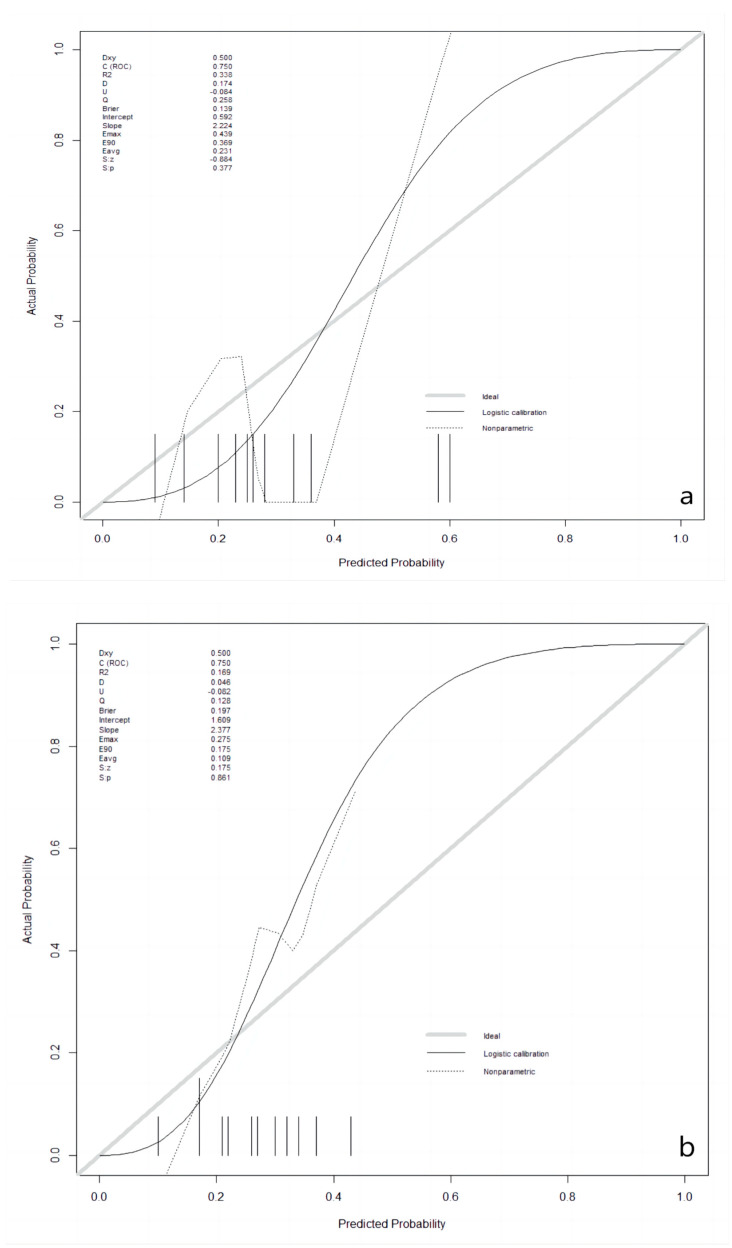
Calibration plots in the validation and the testing datasets. (**a**): in the validation dataset; (**b**): in the testing dataset.

After the best prediction model was defined, the agreement between predictive and true values was examined by calibration plots in the validation and the testing sets, respectively (Figure 2). The results for validation and testing groups from the calibration showed the following: Brier: 0.139 vs. 0.197; *p*: 0.377 vs. 0.861. The Brier score is a metric used to evaluate the performance of predictive models, measuring the difference between the model’s predicted probabilities and the actual observed outcomes. The Brier score ranges from 0 to 1. A lower Brier score indicates better predictive ability of the model, indicating a smaller difference between the model’s predicted probabilities and the actual observed outcomes. When the *p*-value for a calibration curve is greater than 0.05, it typically indicates that there is no significant difference between the observed and predicted values. Our results indicate that there was no deviation between observed and predictive values in the two datasets.

### 3.5. Decision Curve Analysis of the Best Model

**Figure 3:**
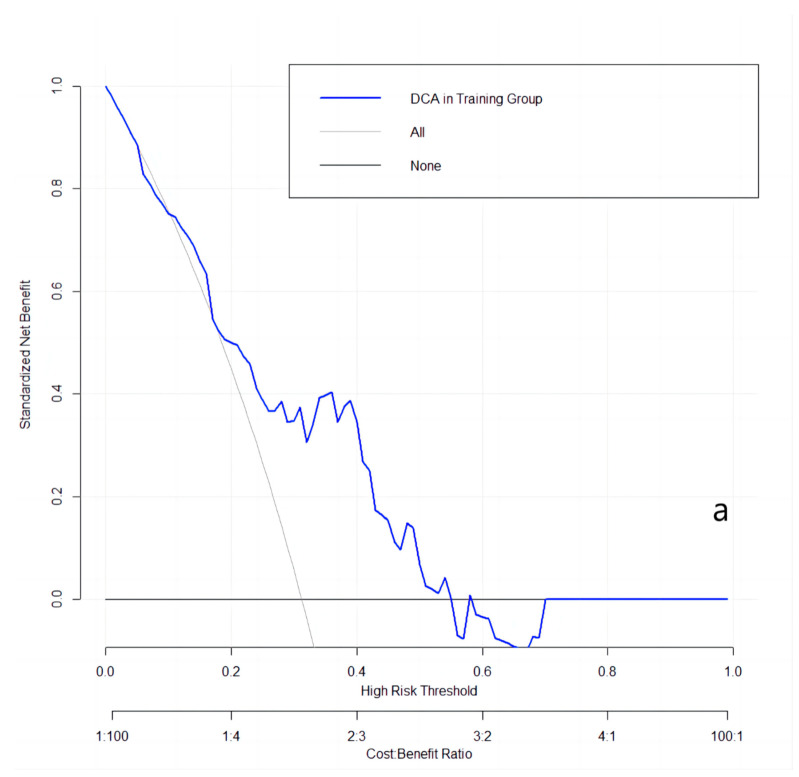

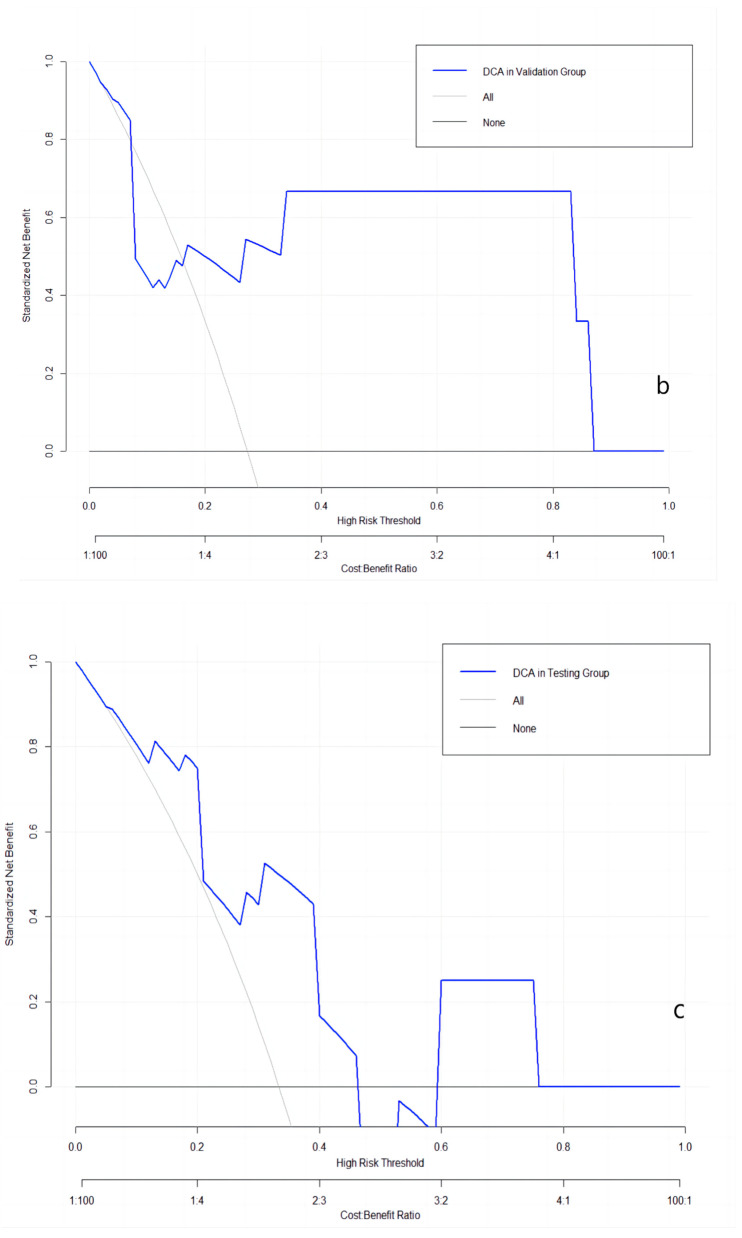
DCA curves in the training, validation, and testing datasets. (**a**): in the training dataset; (**b**): in the validation dataset; and (**c**): in the testing dataset.

The clinical utilities in predicting KRAS mutation of our model were evaluated by DCA curves in the patients from three data groups (Figure 3). In the training set (Figure 3a), when the prevalence of KRAS is between 0.103 and 0.555, our KRAS mutation prediction model showed higher standardised net benefit (sNB, 0.75 to 0) than considering all patients as having a KRAS positive status. An intersection point was found between the curves of our model, and the curve of all patients is KRAS positive (Figure 3b). When the mutant rates of KRAS range from 0.023 to 0.072 (sNB: 0.95 to 0.79) and from 0.162 to 0.870 (sNB: 0.48 to 0), our prediction model showed better clinical utility. Finally, when the prevalence is between 0.054 and 0.468, with the range of sNB 0.86 to 0, our model showed higher usefulness in clinical diagnostics (Figure 3c).

### 3.6. Nomogram of the Best Prediction Model

**Figure 4:**
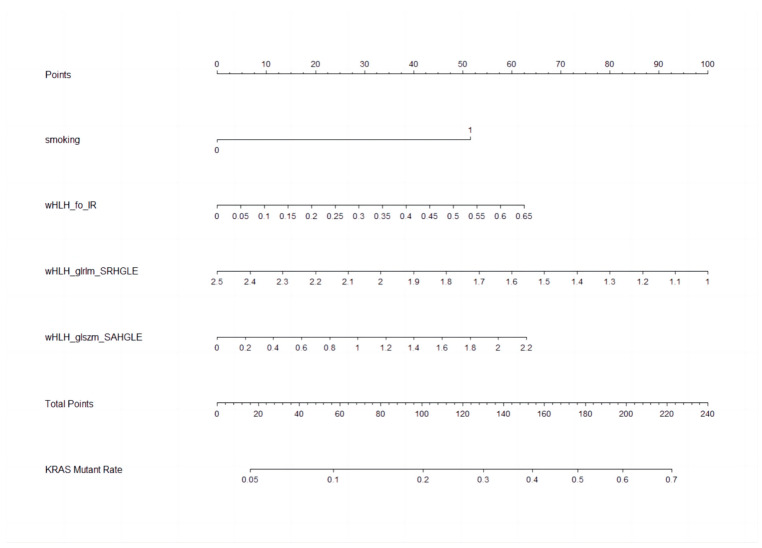
Nomogram to clinically present the prediction model. Smoking: 0 means non-smokers; 1 means smokers. wHLH_fo_IR (WaveletHLH_first order_Interquartile Range); wHLH_glszm_SAHGLE (WaveletHLH_gray level size zone matrix_Small Area High Gray Level Emphasis); wHLH_glrlm_SRHGLE (WaveletHLH_gray level run length matrix_Short Run High Gray Level Emphasis).

The nomogram, a user-friendly and straightforward graphical method with the best predictive capabilities, is presented in Figure 4. The nomogram shows that smoking history, higher wHLH_fo_IR, higher wHLH_glszm_SAHGLE, and lower wHLH_glrlm_SRHGLE obtain the highest scores in predicting KRAS mutation status. The nomogram consists of a total of seven lines. The first line represents the variables included in our predictive model. Specifically, the second line represents the smoking factors, where 0 denotes non-smokers, and 1 indicates smokers. The third to fifth lines are the values of the radiomic features included in the model and their range after normalisation in the current cases. The “Total Points” line represents the sum of scores for all variables. The seventh line represents the KRAS mutation rate, which can be estimated based on the result of the “Total Points”. The final equation is KRAS_mutationrate = −1.6340 + 0.1540 × wHLH_fo_IR − 0.3511 × wHLH_glrlm_SRHGLE + 0.3150 × wHLH_glszm_SAHGLE + 0.9482 × Smoking.

## 4. Discussion

Currently, sotorasib and MRTX849, which target KRAS G12C, have been approved by the Food and Drug Administration (FDA). Clinical trials are underway for MRTX1133, ERAS-4693, and ERAS-5024, which target KRAS G12D [31,32]. Considering the heterogeneity of solid tumours, small biopsies may not reflect the mutational status properly. PET/CT, as an advanced imaging method, can reflect tumour metabolic situations properly and provide additional information in evaluating a tumour. Radiomics extracted from PET images from the whole tumour may contain more details in predicting gene mutational status. Our prediction model based on FDG-PET radiomic features showed good predictive ability in evaluating the KRAS status. In our model, one clinical variable and three PET textural features were included.

Smoking history was highly related to KRAS mutant type in the training group. Our result aligns with previously published studies [11,14,15,16,17], but in validation and testing datasets, smoking history was not associated with KRAS mutation. These negative results may have been caused by the relatively small sample size of two groups and an imbalanced distribution. In the study from Rios et al., the authors built a clinical prediction model to evaluate KRAS mutational status in lung adenocarcinoma. The model contained age, gender, smoking history, race, and clinical stage, showing good performance in predicting (AUC = 0.75) [27].

Three textural features were selected in our prediction model. All of them are based on Wavelet HLH transformation. The first-order statistic features describe the distribution of voxels intensity in medical images (https://pyradiomics.readthedocs.io/en/latest/features.html, accessed on 18 June 2023). The Interquartile Range is the difference between the 75th and 25th percentile of the voxel intensity in images. The large value of the Interquartile Range may reveal the larger distance in the two percentile values, indicating the high heterogeneity in tumours with KRAS mutation. In a study performed on colorectal cancer, 63 radiomic features were extracted from FDG PET/CT images, and the results revealed that the 25th percentile of SUVmax was the independent risk factor for KRAS mutation [20].

At the same time, higher wHLH_glszm_SAHGLE was also positively related to KRAS mutation. GLSZM is a feature family, quantifying grey level zones in an image. A grey level zone is a voxels group with the same grey level. The higher value of GLSZM indicates a higher number of grey level zones in an image, which may lead to a highly heterogeneous tumour. SAHGLE demonstrates the number of small-size zones with higher grey level values in an image (https://pyradiomics.readthedocs.io/en/latest/features.html, accessed on 18 June 2023). Our results indicate that tumours with KRAS mutation tend to contain many small groups with high grey levels. This result resembles a study of pancreatic ductal adenocarcinoma [22]. These authors collected 48 cases and extracted 35 radiological features from FDG PET images. Two textural features, low-intensity zone emphasis and low-intensity larger-zone emphasis from the GLSZM group, were significantly related to KRAS mutation.

Finally, the feature SRHGLE from GLRLM was negatively related to mutated KRAS. The features from GLRLM calculate the length in the number of pixels of how many same grey level values are included in consecutive pixels. SRHGLE measures the joint distribution of shorter run lengths with higher grey-level values (https://pyradiomics.readthedocs.io/en/latest/features.html, accessed on 18 June 2023). In our study, tumours without KRAS mutation showed higher SRHGLE values.

Our prediction model showed high performance in KRAS mutation evaluation in the training, validation, and testing groups, as well as 5-fold cross-validation in the training set. Our result is similar to the study conducted by Shiri et al. in 2020 [25]. They also used the lung radiogenomic dataset from TCIA, and the radiomics abilities were analysed based on FDG PET, low-dose CT, and diagnostic CT. Six different feature selection methods and 12 classifiers were used to build various models. In the FDG PET image set, two models showed the same predictive ability (AUC = 0.8): Log_0.5S + SM + SGD and Wavelet_HHH + SKB + BNB. However, in other studies of FDG PET radiomics in NSCLC, no relationship or predictive performance was found in KRAS mutation [24,33].

Our dataset has an imbalanced distribution in KRAS mutation (mutant/wild type: 37:82 in the whole dataset; 30:66 in the training set; 3:8 in the validation set; and 4:8 in the testing set). According to previous experiences, this kind of imbalanced data needs to be solved by the Synthetic Minority Over-sampling TEchnique (SMOTE) [34]. However, in our procedure for model building, we did not use SMOTE to balance our dataset. That has two reasons: first, our predictive model showed better performance without SMOTE, and second, it was reported that the KRAS mutation rate in lung adenocarcinoma is about 26.1% [6]. In our training group, the KRAS mutation rate was 31.25%, which was close to the expectable rate. Hence, we chose the original dataset to build and validate our model instead of using SMOTE.

In addition, except for the validation group, we also chose a 5-fold CV and a testing group to check the predictive performance and general usability of our model. Limited by the small training sample size, we took a 5-fold CV as the first validating method after modelling, which also enlarged our sample size. The following phenomenon was observed in our study: even if a model showed good predictive performance in the training set, 5-fold CV, and the validation dataset, it was not easy to achieve the same ability in the new testing group. Thus, we thought the testing group was essential to ensure that good performance was not obtained accidentally.

There are several limitations in our current study. First, this was a retrospective study, limited by its nature; second, although we took the publicly available dataset to enlarge our sample size, the number of cases in our study was still relatively small; third, we put all samples together and split them randomly, meaning that the FDG PET data were obtained from different scan devices and different scan protocols.

## 5. Conclusions

Our retrospectively established model shows high performance in predicting KRAS mutation status in lung adenocarcinoma. However, it still needs validation in larger prospective studies in the future.

## Figures and Tables

**Table 1:** The comparison of clinical data among three datasets.

Characteristics	Training Set		*p*	Validation Set		*p*	Testing Set		*p*
	KRAS (−)	KRAS (+)		KRAS (−)	KRAS (+)		KRAS (−)	KRAS (+)	
	*n* = 66	*n* = 30		*n* = 8	*n* = 3		*n* = 8	*n* = 4	
Age, *n* (%)			0.826			1.000			0.491
≤69	34 (52%)	14 (47%)		4 (50%)	2 (67%)		5 (63%)	4 (100%)	
>69	32 (48%)	16 (53%)		4 (50%)	1 (33%)		3 (38%)	0 (0%)	
Gender, *n* (%)			1.000			1.000			0.546
male	36 (55%)	17 (57%)		6 (75%)	3 (100%)		3 (38%)	3 (75%)	
female	30 (45%)	13 (43%)		2 (25%)	0 (0%)		5 (63%)	1 (25%)	
Smoking History, *n* (%)			0.042			0.491			0.491
no	21 (32%)	3 (10%)		3 (38%)	0 (0%)		3 (38%)	0 (0%)	
yes	45 (68%)	27 (90%)		5 (63%)	3 (100%)		5 (63%)	4 (100%)	
Pleural Invasion, *n* (%)			0.397			1.000			1.000
no	49 (74%)	19 (63%)		6 (75%)	2 (67%)		5 (63%)	2 (50%)	
yes	17 (26%)	11 (37%)		2 (25%)	1 (33%)		3 (38%)	2 (50%)	
Diameter, *n* (%)			0.048			1.000			1.000
≤18.05	38 (58%)	10 (33%)		4 (50%)	1 (33%)		4 (50%)	2 (50%)	
>18.05	28 (42%)	20 (67%)		4 (50%)	2 (67%)		4 (50%)	2 (50%)	

KRAS: Kirsten rat sarcoma virus.

**Table 2:** Performance of three prediction models in three datasets.

Groups	Models	AUC (95% CI)	Accuracy	Sensitivity	Specificity	PPV	NPV
**Training Set**	Model 1	0.682 (0.578, 0.785)	0.667	0.600	0.697	0.474	0.793
	Model 2	0.731 (0.619, 0.843)	0.750	0.667	0.788	0.588	0.839
	Model 3	0.694 (0.576, 0.811)	0.708	0.533	0.788	0.533	0.788
	**5-fold CV**						
	Model 1	0.672		0.915	0.091		
	Model 2	0.686		0.892	0.239		
	Model 3	0.655		0.929	0.206		
**Validation Set**	Model 1	0.708 (0.408, 1.000)	0.545	1.000	0.375	0.375	1.000
	Model 2	0.750 (0.248, 1.000)	0.909	0.667	1.000	1.000	0.889
	Model 3	0.667 (0.201, 1.000)	0.727	0.667	0.750	0.500	0.857
**Testing Set**	Model 1	0.656 (0.355, 0.957)	0.583	1.000	0.375	0.444	1.000
	Model 2	0.750 (0.448, 1.000)	0.667	1.000	0.500	0.500	1.000
	Model 3	0.688 (0.349, 1.000)	0.583	1.000	0.375	0.444	1.000

ROC: Receiver Operating Characteristic; AUC: Area Under the ROC Curve; 95%CI: 95% Confidence Interval; PPV: Positive Predictive Value; NPV: Negative Predictive Value; and CV: cross-validation.

## Data Availability

The data presented in this study are available on request from the corresponding author.
